# Synergy as a rationale for phage therapy using phage cocktails

**DOI:** 10.7717/peerj.590

**Published:** 2014-09-25

**Authors:** Matthew Schmerer, Ian J. Molineux, James J. Bull

**Affiliations:** 1 Center for Computational Biology and Bioinformatics, The University of Texas at Austin, Austin, TX, USA; 2 Department of Molecular Biosciences, The University of Texas at Austin, Austin, TX, USA; 3 Institute for Cellular and Molecular Biology, The University of Texas at Austin, USA; 4 Department of Integrative Biology, The University of Texas at Austin, USA

**Keywords:** Phage therapy, Models, Dynamics, Depolymerase, Dynamics, Bacteria, Biofilm

## Abstract

Where phages are used to treat bacterial contaminations and infections, multiple phages are typically applied at once as a cocktail. When two or more phages in the cocktail attack the same bacterium, the combination may produce better killing than any single phage (synergy) or the combination may be worse than the best single phage (interference). Synergy is of obvious utility, especially if it can be predicted *a priori*, but it remains poorly documented with few examples known. This study addresses synergy in which one phage improves adsorption by a second phage. It first presents evidence of synergy from an experimental system of two phages and a mucoid *E. coli* host. The synergy likely stems from a tailspike enzyme produced by one of the phages. We then offer mathematical models and simulations to understand the dynamics of synergy and the enhanced magnitude of bacterial control possible. The models and observations complement each other and suggest that synergy may be of widespread utility and may be predictable from easily observed phenotypes.

## Introduction

A common practice in phage therapy of bacterial infections is the administration of several phages at once, in mixtures or cocktails (Merabishvili et al., 2009; Gill & Hyman, 2010; Chan, Abedon & Loc-Carrillo, 2013). One obvious advantage of a cocktail is a large collective host range that may obviate the need to characterize phage sensitivities of the infecting pathogenic bacteria. A second possible advantage is in thwarting resistance: if multiple phages target the same bacterium, evolution of resistance to all such phages may be required before treatment fails. A third possible mechanism is dynamical: two phages may collectively kill the bacterial population more rapidly or more completely than either phage alone. This latter process of ‘synergy’ between phages is relatively unexplored, perhaps because its demonstration requires quantitative assessment of bacterial densities during treatment. To the extent that single phage types may be insufficient for resolving infections, synergy offers a potential tool for improving phage therapy. It is also a process whose foundations are intrinsically dynamic and may thus benefit from specific study to identify principles.

In the type of synergy considered here, the growth of one phage *augments* the infection properties of a second phage on the same bacterium (which may be contrasted with two phages having somewhat *complementary* host ranges). This augmentation can operate in one direction or both. For a lytic phage, growth is primarily determined via effects on any of three properties (Adams, 1959): the rate the phage infects cells (adsorption rate), the number of progeny per infection (burst), and the time from infection until progeny are released (lysis time or latent period). In theory, synergy could operate via effects on any of them, but documented examples are so far restricted to effects on infection rate: one phage produces a depolymerase that strips the bacterial capsule (Sutherland, 1995; Hughes et al., 1998; Azeredo & Sutherland, 2008) enhancing infection by another phage (Bull, Vimr & Molineux, 2010). Phages can also interfere with each other, as is well documented at the intracellular level in which coinfection by two phages reduces the burst size of one (Delbruck, 1945; Adams, 1959).

Understanding phage synergy presents challenges at two levels. Synergy may require specific molecular mechanisms enabling one phage to augment the growth of another. Yet even when the molecular mechanisms conspire to synergize, the population dynamics of phage and bacteria may operate against both phages having much greater killing than either does alone. If the population dynamics works against two phages killing far better than one, for example by the rapid loss of one phage, there is no practical benefit of synergy. Ultimately, synergy will be most useful if it is robust and can be predicted from easily observed properties that do not require intensive, mechanistic study. Our chief goal is to explore that perspective.

## Results

### A demonstration of synergy

The meaning of synergy applied here requires that one phage augments the growth properties of a second phage. A biological context for this augmentation is tailspike enzymes. Phage tailspikes commonly carry enzymes that degrade extracellular carbohydrates produced by bacteria, enhancing access to the cell surface (Sutherland, 1995; Hughes et al., 1998; Azeredo & Sutherland, 2008). After cell lysis, a substantial amount of tailspike enzyme that had been synthesized is likely not to be assembled on progeny phage, but be released as a free enzyme. When these phages are plated under suitable conditions, the diffusing enzymes may generate plaque halos that expand over time (Bessler et al., 1975; Cornelissen et al., 2011; Kassa & Chhibber, 2012). If one phage can improve its adsorption rate by exploiting the enzyme released by another, the combined killing by both phages may exceed that by either alone.

This context leads naturally to a terminology that is adopted here. The phage producing the shared enzyme is referred to as the *donor* phage, and the phage benefitting from the enzyme is referred to as the *recipient* phage.

#### Isolation of a colanidase-producing phage as a candidate donor

**Figure 1:**
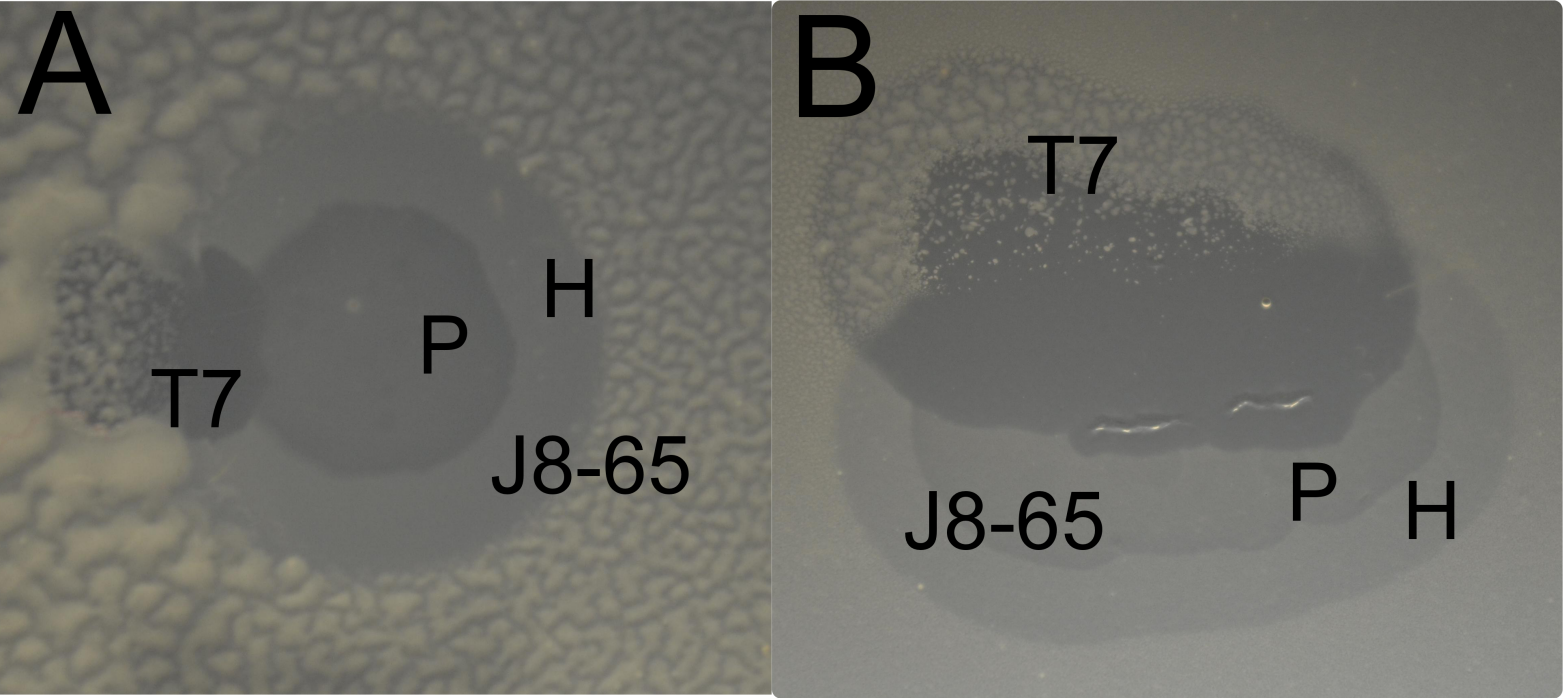
Two examples of a halo of J8-65 invaded by T7 (A and B). Enhanced clearing is observed where the T7 plaque intersects both the halo and plaque of J8-65. Note the growth of mucoid colonies in the regions infected by T7 alone. (Bacterium IJ2308 was spread on plates without top agar, and suspensions of both phages were spotted close enough to each other that the expanding plaques would intersect during overnight growth. In contrast to the assays of cell killing, these plates were incubated longer (37 °C overnight followed by 24 h at room temperature) to enhance the visibility of mucoid colonies.) P, plaque of J8-65; H, halo of J8-65; T7, plaque of T7.

When screening sewage for phages that degrade colanic acid, we observed a few large clear plaques on lawns of a mucoid *E. coli* (IJ2308). Attempts to purify an isolate that also formed large plaques led to the discovery that the large plaque phenotype resulted from a combination of two phages. One phage was apparently closely related to T7, possibly of lab origin; the other was a wild phage (J8-65) which, when plated on IJ2308 in glucose-containing media forms large, somewhat turbid plaques with much larger expanding halos (Fig. 1), although both plaque size and halo properties depend on media and temperature. The genome sequence of J8-65 reveals that it is closely related to *Pantoea agglomerans* phage LIMEzero, a member of the *ϕ*KMV-like viruses (Adriaenssens et al., 2011) in the *Podoviridae*. J8-65 encodes a gene with 87% amino acid identity to a well-characterized colanidase (Firozi et al., 2010), and this enzyme is likely the basis of the observed expanding halos.

#### A test of synergistic killing

**Figure 2:**
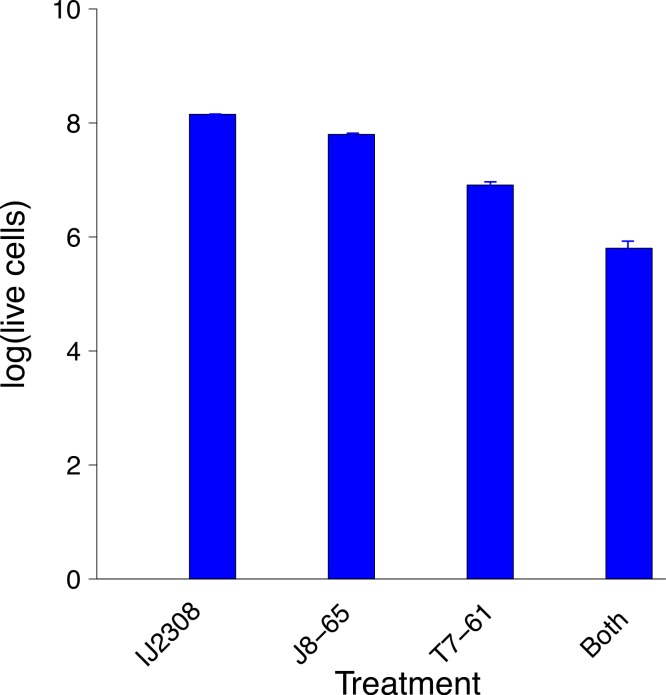
Synergistic reduction of bacteria by two phages. The leftmost bar gives the average number of IJ2308 cells per plate, untreated. The next two bars give the numbers of viable cells from plates treated with single phages. The rightmost bar is from plates treated with both phages. There is statistically significant heterogeneity across all treatments (*P* ≪ 0.0001), and treatment with both phages is significantly lower than T7 alone (*P* ∼ 0.0012). Data were obtained by flow cytometry and logged before doing statistics. Error bars show 1 std. error, often too small to see. The vertical scale is log_10_ of live cell density.

Synergy was anticipated from the casual observation that T7 forms expanded clearings on IJ2308 if one of its plaques grows into a halo or plaque of J8-65 (Fig. 1). This behavior suggested that T7 is the recipient, J8-65 the donor. The existence and magnitude of synergy was formally tested by plating a high density (≈ 10^5^ pfu) of the two phages both individually and together with host IJ2308 on M9 glucose and counting viable cells using flow cytometry (Fig. 2). The combination of both phages results in a 10-fold greater killing than with T7 alone and nearly 100-fold greater than with J8-65 alone.

Phage dynamics during synergy were inferred from titers after overnight growth on the plate. The net amplifications were different for the two phages: The T7 titer increased more than 10,000-fold from its inoculum, J8-65 grew just over 300-fold. Thus, T7 amplified about 50-fold more than did J8-65. As will be shown below in simulations, unequal amplification of each phage is expected under synergy with appropriate starting densities. Indeed, higher amplification by the recipient phage is evidence that the donor and recipient phages were chosen appropriately and were administered at suitable relative densities.

The poor killing by J8-85 alone likely reflects a general inability to infect most cells. The 1-log killing by T7 alone stems from its effective killing to generate small plaques but an inability to kill beyond zones of high phage concentration, possibly due to bacterial protection by colanic acid as the lawn matures. (In Fig. 1, the plaque areas of T7 alone are populated with mucoid colonies, indicating an inability of T7 to invade those cells.) The synergy may thus stem from J8-65 colanidase activity increasing T7 access to the bacterial cell surface. That there is not a greater combined killing than 2 logs is due to some regions of the plate not being accessed by either or both phages (which is clearly dependent on the densities plated). We commonly observed that although T7 plaques were large in the presence of J8-65, they did not achieve confluence across the plate.

#### Mechanism

**Figure 3:**
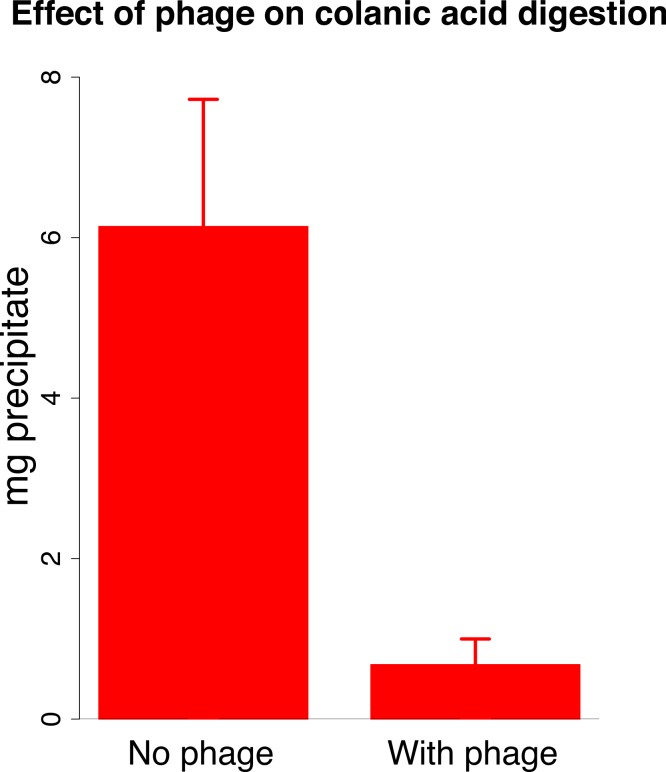
Effect of phage J8-65 on high molecular weight colanic acid components after overnight growth on plates. M9 glucose plates were spread with IJ2308 or IJ2308 plus J8-65 and incubated overnight at 37 °C. The high molecular weight (MW) fraction was recovered as a congealed precipitate in the absence of centrifugation. As shown in Fig. 2, this phage causes little reduction in live bacterial counts. Error bars represent 1 standard error, shown for each fraction.

The mechanism of synergy likely lies with the J8-65 colanidase degrading the mucoid surface layer and improving access of T7 to its receptor. However, our data thus far are only of cell counts, and evidence of synergistic killing is compatible with other mechanisms. Two further lines of evidence were sought on the mechanism of synergy. First, we observed that expanded clearing by T7 begins at the edge of the J8-65 halo (Fig. 1), whereas J8-65 phage could not be detected in stabs taken from the halo periphery. This observation indicates that the benefit to T7 is due to the contribution of components from, or an effect of, J8-65 instead of from intact phages. Second, J8-65 reduces the extent of high molecular weight colanic acid on plates. The amount of high molecular weight colanic acid (and other soluble polysaccharides) was measured on M9 glucose plates of IJ2308 either seeded with J8-65 or without phage. Soluble carbohydrates were obtained from washes of the plate surface after overnight growth; the high molecular weight (MW) component was obtained as a precipitate formed in suspension after addition of acetone. Addition of J8-65 caused a marked and statistically significant reduction in the high MW component (Fig. 3).

### A mathematical model of dynamics with synergy

#### Intuition often fails

Many aspects of phage-bacterial dynamics are unintuitive. Not only does phage growth depend on 3 parameters, but rapid phage growth requires moderate to high bacterial densities. Yet phage-induced cell lysis reduces the bacterial density on which that rapid growth depends. Mathematical models can greatly facilitate understanding otherwise unintuitive dynamics. In the case of synergy, two puzzles specifically motivate our use of models. First, synergy presumably requires a high density of the donor phage to augment the recipient phage. Yet if the donor is able to attain high density, why is the donor phage alone not sufficient to control the bacteria? Second, does synergy require the dynamical maintenance of both phages to be effective? If so, how can both phages can be maintained, as two different phages do not typically have equal growth rates? More generally, we are interested in whether synergism obeys dynamical properties that are broadly generalizable and might be inferred *a priori* from easily observed phenotypes.

Insight to these questions will be provided by mathematical models. For simplicity, only obligately lytic phages will be considered. Before proceeding to the formal model, we offer a perspective on the second puzzle—how two phages can be maintained indefinitely. It will generally be true of any two phages growing on the same host population that one phage will intrinsically outgrow the other in the absence of synergy. However, synergy alters the dynamics by increasing the growth rate of one phage in the presence of the other. This interdependence between the phages is the key to the maintenance of both, but the interdependence alone does not ensure that both phages are maintained.

Maintenance of both phages requires that both have the same net growth rate over the long term. This becomes possible if the growth rate of the recipient phage is intrinsically lower than that of the donor phage but then increases as the donor phage becomes common. To avoid loss, the recipient phage growth rate must surpass the donor’s growth rate when and only when the donor phage is at high density. In this way, the recipient phage can outgrow the donor phage but cannot displace it, as the recipient phage will once again become inferior when the donor phage declines.

**Figure 4:**
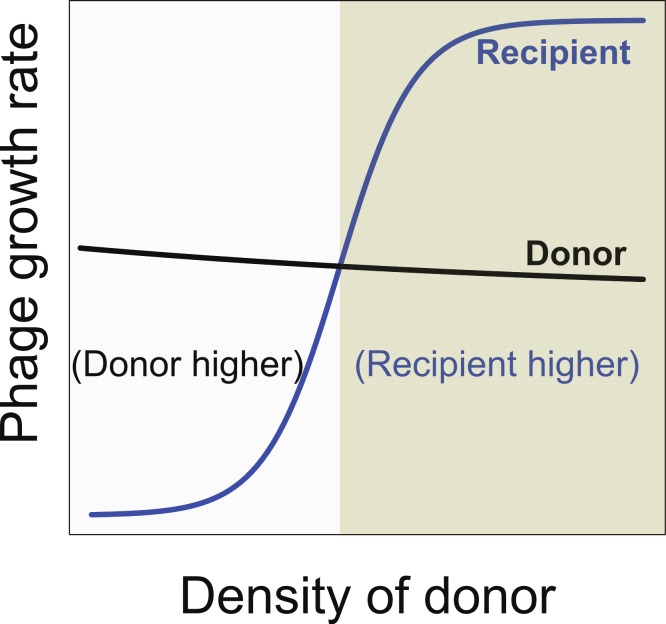
Necessary conditions for the long term co-maintenance of two phages (donor and recipient). For convenience, the growth rate of the donor phage is drawn as a curve with a shallow slope. The important condition for maintenance is that the growth rate of the recipient phage be lower than that of the donor phage when it is at low density, but surpass that of the donor phage when at high density. This property ensures that the recipient phage cannot replace the donor phage but that if the donor phage attains high density, the recipient phage will overtake it. Areas are differentially shaded to distinguish the region in which the donor has the higher growth rate from that in which the recipient has the higher growth rate.

Therefore, a necessary condition for synergy to enable maintenance of two phages is that synergy reverse the relative growth rates of the two phages (Fig. 4). At low density, the donor phage outgrows the recipient, but at high donor density and maximal synergy, the recipient phage outgrows the donor. However, this condition is still insufficient, because the equilibrium bacterial density must also lie in the range at which the recipient phage growth rate is higher than that of the donor phage. Whether the equilibrium bacterial density is high enough for maintenace of both phages is not easily comprehended without modeling.

#### Formal dynamics when both phages are maintained together

We offer a differential equation model of phage bacterial dynamics (Eq. (1)), similar to those in Levin, Stewart & Chao (1977) and more recently Bull et al. (2014). The model describes the numbers (densities) over time of phage and bacteria in an environment with continuous flow, such as a chemostat. The continuous flow is represented as a constant washout/death rate of all variables. The model assumes mass action, as in liquid (an assumption that renders the model tractable and its results intuitive). A model assuming spatial structure would be more appropriate for some contexts, but such models are unwieldy and do not yet readily lend themselves to easy interpretation.

The model has 7 equations that accommodate two phages: the donor phage (density *P_D_*) produces a freely diffusible substance (density *S*) enhancing the adsorption rate of the recipient, and the recipient phage (density *P_R_*), whose adsorption rate depends on *S*. The substance is released at lysis by the donor phage in a manner similar to the release of phage progeny. The adsorption rate function of the recipient phage (*k*(*S*)) has a minimum value of 10^−12^ when *S* = 0, increasing with *S* up to 10^−9^ mL/min.

Parameters and variables are defined in Table 1. In the absence of phage, bacterial growth obeys a logistic function with carrying capacity *C*. Lysis is modeled as a delay function *L* minutes after infection, hence a subscript *L* indicates the value of the variable *L* minutes in the past. A superior dot ($\dot {}$) indicates a derivative with respect to time:

(1) \begin{eqnarray*} \begin{array}{l} \displaystyle \dot {B}=v\left(1-\frac{B}{C}\right)B-({k}_{D}{P}_{D}+k(S){P}_{R})B-w B\\ \displaystyle {\dot {P}}_{D}={b}_{D}~{\mathrm{e}}^{-w L}{k}_{D}{P}_{{D}_{L}}{B}_{L}-{k}_{D}{P}_{D}\left(B~+~{I}_{D}+{I}_{R}\right)-w{P}_{D}\\ \displaystyle {\dot {P}}_{R}={b}_{R}~{\mathrm{e}}^{-w L}k({S}_{L}){P}_{{R}_{L}}{B}_{L}-k(S){P}_{R}\left(B~+~{I}_{D}+{I}_{R}\right)-w{P}_{R}\\ \displaystyle {\dot {I}}_{D}=-{\mathrm{e}}^{-w L}{k}_{D}{P}_{{D}_{L}}{B}_{L}+{k}_{D}{P}_{D}B-w{I}_{D}\\ \displaystyle {\dot {I}}_{R}=-{\mathrm{e}}^{-w L}k({S}_{L}){P}_{{R}_{L}}{B}_{L}+k({S}_{L}){P}_{R}B-w{I}_{R}\\ \displaystyle \dot {S}=Z{\mathrm{e}}^{-w L}{k}_{D}{P}_{{D}_{L}}{B}_{L}-w S\\ \displaystyle k(S)=1{0}^{-12}(1000-999{~\mathrm{e}}^{-1{0}^{-7}S}). \end{array} \end{eqnarray*}

**Table 1:** Model variables and parameters.

Notation	Description	Values
**Variables**		
*B*	Density of uninfected bacteria	
*P_D_*	Density of free donor phage	
*P_R_*	Density of free recipient phage	
*S*	Density of substance produced by donor phage that increases adsorption rate of the recipient phage	
*I_D_*	Density of bacteria infected with donor phage (before lysis)	
*I_R_*	Density of bacteria infected with recipient phage (before lysis)	
**Functions**		
k(S)	Adsorption rate of recipient phage (mL/min)	10^−12^(1000−999 e^−10^−7^*S*^)
**Parameters**		
*k_D_*	Adsorption rate of donor phage (mL/min)	5 × 10^−11^
*w*	Washout/death rate (/min)	0.1
*b_D_*	Burst size of donor phage	50
*b_R_*	Burst size of recipient phage	300
*L*	Lysis time (min)	25
*v*	Maximum bacterial growth rate (/min)	0.35
*Z*	Production rate of *S* from a single burst	1
*C*	Carrying capacity of environment	5 × 10^9^

**Figure 5:**
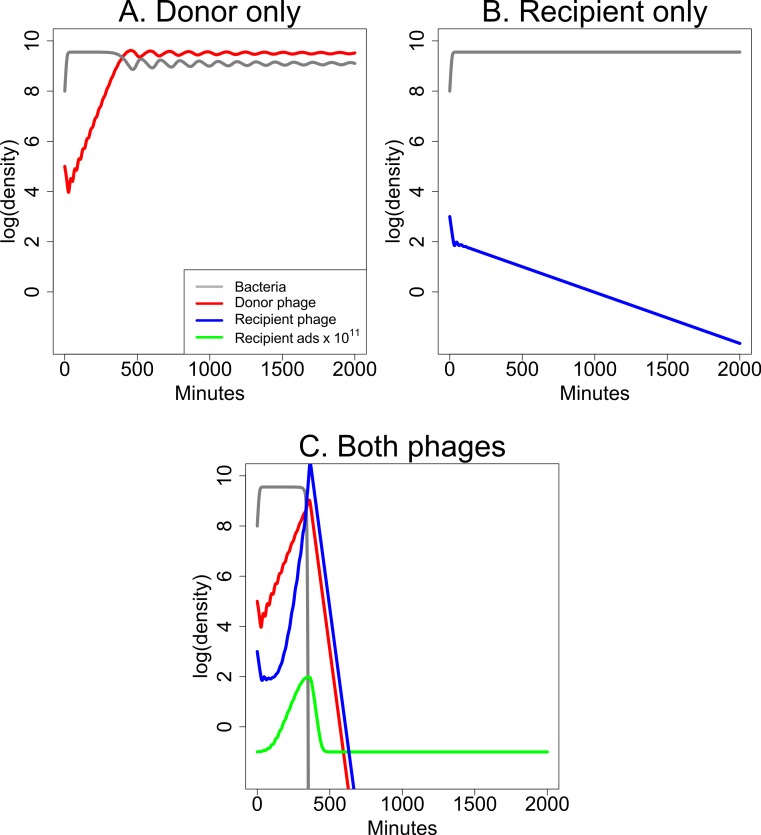
Dynamics of donor and recipient phages separately and together that illustrate synergy. (A) The donor phage has a low enough adsorption rate and burst size that, although it is maintained, it does not suppress bacterial densities appreciably. (B) The recipient phage growth properties are too poor for it to be maintained by itself, so bacterial densities remain high. (C) Synergy: the combination of the two phages from (A) and (B) leads to a profound decline in bacterial densities even though the growth parameters are unaltered from the panels of single phages. Extending the time scale would reveal ongoing oscillations in phage and bacterial densities, as is typical for these models (Levin, Stewart & Chao, 1977); both phages are maintained indefinitely. The legend in (A) applies to all three panels; the green curve for the recipient adsorption rate (× 10^11^) is 10^11^ times the *k*(*S*) value. Phage growth parameter values are given in Table 1. The vertical axis uses a scale of log_10_.

Short-term dynamics are illustrated in Fig. 5 for a specific set of parameter values. The dynamics continue indefinitely, and they invariably show ongoing oscillations, typical of predator–prey dynamics, because the growth of each species depends on the density of the other. The first cycle of phage suppression of bacterial densities (as shown) is presumably most relevant to therapeutic treatment because the initial drop in bacterial density should determine whether the infection is brought under control. The figures compare the effects of single phages (Figs. 5A and 5B) with the effects of both phages combined (Fig. 5C). Figure 5A is of a culture with bacteria and donor phage; it shows the ‘paradoxical’ outcome in which a single phage and bacteria coexist at high density. Figure 5B is of the recipient phage and bacteria; the phage is lost due to poor infection parameters. Figure 5C reveals synergy when both phages are included, the recipient phage adsorption rate benefitting from *S*, produced by the donor phage.

Although the model is quantitative, its purpose is to expose critical factors that underlie general dynamical properties. The model thus does not attempt to capture explicit details of any empirical system but instead captures properties that should apply to many systems. The parameters were chosen to satisfy properties that intuition suggests enable synergy. For example, it is known from prior theory that phages with low adsorption rates (but with other suitable growth properties) can grow to high densities but fail to depress bacterial numbers (Levin, Stewart & Chao, 1977)—explaining the riddle of how a phage can reach high densities without suppressing bacterial numbers. These growth characteristics seem appropriate for candidate donor phages and were chosen here. Likewise, the recipient phage must have poor infection properties (a low adsorption rate) by itself but realize profoundly more efficient adsorption when the donor is present. Furthermore, its growth characteristics in the presence of the donor phage must surpass that of the donor for synergy to have a major effect.

We suggest that these conditions are met in the synergy between J8-65 and T7. Thus T7 has outstanding growth characteristics on *E. coli* when it can adsorb efficiently (Bull & Molineux, 2008). The small plaques formed by T7 on IJ2308 suggests that the mucoidy inhibits adsorption. The somewhat turbid plaques formed by J8-65 alone indicate that it does not grow well on IJ2308 by itself; in conjuction with the bioinformatics data, the large halos suggests that J8-65 produces an enzyme that degrades the surface carbohydrates of mucoid cells. The enhanced clear plaque growth of T7 in halos and plaques of J8-65 suggest that J8-65 is augmenting T7 with its enzyme.

The dynamics in this model continue indefinitely; they invariably show oscillations because of the reciprocal density dependence of phage and bacteria—unless of course phage(s) are lost or are largely ineffective (as in Fig. 5B). These oscillations occur because the models are deterministic, and bacterial or phage densities never reach 0, so when phage densities drop to low levels, there are invariably bacteria present that can grow again to high density. Figure 5 is limited to short term dynamics, which are sufficient to show whether phages can have a rapid and profound effect on the bacterial population.

#### Sensitivity

Synergy in this model is at least moderately robust to variations in parameter values. Adsorption rate of the recipient phage (*k*(*S*)) is the basis of synergy, so quantitative changes in that function are of greatest interest. Multiplying or dividing the *k*(*S*) function by 10 retains strong synergy; the 10-fold increase enables the recipient phage to be maintained in the absence of the donor, but the impact of the recipient phage on bacterial densities is nonetheless far greater when the donor is present. The exponent (−10^−7^*S*) reflects the efficacy of *S* in augmenting the recipient phage. Changing it to (−10^−8^*S*) reduces the efficay of *S* but still retains a profound synergy, albeit one from which bacterial densities rebound sooner. The functional form of synergy in this model is necessarily hypothetical, so these simulations merely illustrate that synergy is feasible over broad parameter ranges, hence is a plausible interpretation of the empirical results.

#### Dynamics when one phage is not maintained

**Figure 6:**
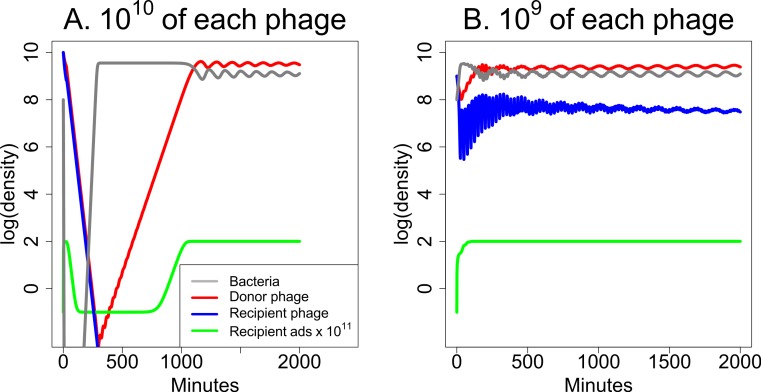
Weak synergy: short term dynamics of donor and recipient phages when synergy is insufficient to maintain both phages. Here the recipient phage is ultimately lost in both (A) and (B). For a pronounced benefit of synergy, the initial dose must be high for both phages—high for the donor phage so it creates a high level of *S* to augment the recipient phage, and high for the recipient phage before it is lost. (A) Initial densities are 10^10^ of both phages, and there is an appreciable clearing of bacteria before the recipient phage is lost. Bacterial densities rebound quickly, however, and remain high thereafter. (B) A mere 10-fold reduction in initial phage densities eliminates substantial killing of the bacteria. The parameters used here are the same as in Fig. 5 except that the burst size of the recipient phage is reduced to 31 from 300. The legend in (A) also applies in (B). The vertical axis uses a scale of log_10_.

The parameters used for Fig. 5 allow both phages to be maintained together indefinitely. However, the parameters can be modified to intensify or reduce synergy or to improve the ability of either phage to kill bacteria in the absence of the other phage. Of great interest is whether synergy remains effective when only one phage is maintained in the long term. The analysis in Fig. 6 suggests that synergy can be achieved when only one phage is maintained, but high phage inocula may be needed to achieve the benefits.

### Interference

The interaction between two phages need not improve treatment. A minor mathematical modification of the previous model generates an outcome in which the donor phage inhibits the recipient phage. The adsorption rate now decreases with *S* instead of increasing:

(2) \begin{eqnarray*} \displaystyle k(S)=\frac{1{0}^{-9}}{1000-999{\mathrm{e}}^{-1{0}^{-7}S}}.&&\displaystyle \end{eqnarray*}

**Figure 7:**
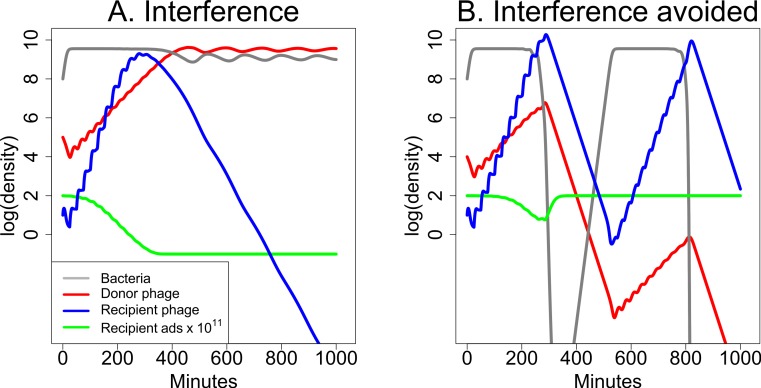
Dynamics of donor and recipient phages with interference, the opposite of synergy. The donor phage has burst size 50 with a fixed adsorption rate of 5 × 10^−11^ and produces a substance (*S*) that reduces recipient phage adsorption. The recipient phage has burst size 100 but has an adsorption rate that declines with *S*:10^−9^/(1000−999e^−10^−7^*S*^). Both (A) and (B) use the same parameters, differing only in the initial abundances of the two phages. (A) The donor phage is initially 10^4^-fold more abundant than the recipient phage, and it is sufficiently able to suppress adsorption by the recipient phage that the recipient phage is lost. (B) The donor phage is initially 10^3^-fold more abundant than the recipient phage, and with this 10-fold relative improvement in initial density (compared to (A)), the recipient phage now overtakes the donor phage before the donor can drive it extinct. In both (A) and (B), the green curve shows 10^11^-fold the adsorption rate of the recipient phage. The legend in (A) also applies to (B). The vertical axis uses a scale of log_10_.

The effect of this modification on bacterial dynamics now shows a major effect of starting conditions (Fig. 7). In the absence of the donor phage, the recipient phage is intrinsically superior—it has both a larger burst and higher adsorption rate than the donor phage, and if it starts at high enough frequency, it will displace the donor phage or prevent its ascent in the total phage population. But if the donor phage frequency reaches a high enough level, its depression of recipient phage adsorption rate causes the recipient phage to be lost. Note that this process of interference is mechanistically distinct from the depressor effect of Delbruck (1945).

## Discussion

As used here, synergy is a dynamical phenomenon in which greater bacterial killing is achieved by two phages than by either phage alone. Our specific focus was synergy, where one phage improves or augments the growth properties of a second phage. Modeling indicates that the synergistic effect can be profound—each phage alone has little effect in controlling the bacteria but together they cause the bacterial population to plummet. The experimental system introduced here, using a mucoid *E. coli*, phage T7 and an uncharacterized wild phage expressing a colanidase, supported the model qualitatively by revealing a greater killing from both phages combined than by either alone.

The conditions in which synergy has such a profound effect are seemingly restrictive. The donor phage, the one augmenting the other (recipient) phage, must kill bacteria well enough by itself to attain high density yet it must not kill so well that it alone depresses the bacterial density. The donor phage must also produce a product or otherwise modify the environment to improve killing by the recipient phage. Finally, once augmented, the recipient phage must greatly outgrow the donor phage. Furthermore, synergy is not a necessary or even likely outcome of mixed infections: interactions among phages may even be antagonistic such that two phages are worse together than the better one is alone. At the same time, we have not systematically explored the conditions conducive to synergy and have restricted consideration to one-way or asymmetric synergy. It should therefore be considered that synergy may operate under a much wider range of conditions than identified here.

Synergy provides an obvious benefit to phage therapy using phage cocktails if it can be orchestrated—which may often require identifying at least the potential for synergy *a priori*. *A priori* recognition of synergy might have seemed a daunting task, so it is especially encouraging that it was possible to anticipate synergy in the experimental system here from an easily observed phenotype: a much enhanced clearing of bacterial lawns when plaques of the two phages merge (Fig. 1). Furthermore, little experimentation was necessary to confirm the synergistic nature of the initial observation. Of the two phages used here, one (T7) was well characterized in advance of the study but the other was poorly studied. It is not even known if *E. coli* K-12 is an optimal or even a good host for J8-65. It remains to be seen how easily synergy can be predicted when both phages are uncharacterized, as will often be the case in treatment. Perhaps the intersecting plaque phenotype, or a more rapid clearing of a liquid culture, is sufficient to tentatively identify synergistic interactions.

Just as phage infection dynamics are sensitive to environmental details, so synergy is likely to be sensitive. Populations of bacteria may be heterogeneous with respect to phage susceptibility; heterogeneity may be spatial, temporal or stochastic, due to variation in gene expression induced by temperature, resources, substrate and bacterial age. In the present study, plaque phenotypes on mucoid hosts were sensitive to media, temperature and strain. As synergy depends on the infection dynamics of two phages, it may be especially sensitive to bacterial variation affecting the dynamics of single phages. Whether synergy will prove robust and prevail amid bacterial variation awaits comprehensive empirical study.

Intrinsic phage dynamics do not necessarily work in favor of maintaining synergistic combinations: one phage may outgrow and displace the other despite synergy. One resolution of this problem in therapeutics would be to apply high enough densities of the phages that any synergistic benefits are realized before intrinsic dynamics ensue. Alternatively, treatment could combine the recipient phage with just the product of the donor phage that was responsible for the synergy.

The examples discussed here are of unidirectional synergy, with one donor phage and one recipient. Bi-directional synergy may be an especially useful option with biofilms. It is well appreciated that phage depolymerase enzymes enhance phage access to biofilms (Hughes et al., 1998; Hanlon et al., 2001; Lu & Collins, 2007; Azeredo & Sutherland, 2008; Cornelissen et al., 2011). If the biofilm extracellular matrix is comprised of multiple carbohydrates, and different depolymerases digest different biofilm components, the combination of phages with different depolymerases may more fully eradicate the biofilm than could any single phage.

A precedent for synergy was reported between phages infecting a K1-capsulated *E. coli* (Bull, Vimr & Molineux, 2010). The growth rate of a phage whose bacterial receptor was presumably the O-antigen was enhanced by the addition of a tailspike enzyme of a different phage that degraded the K1 capsule. In contrast to the J8-65 x T7 synergy here, there was no practical benefit of synergy in that system because the recipient phage growth was no better than that of the candidate donor phage.

An understanding of synergy provides an obvious motivation for genome engineering of phages. When the donor phage provides a single gene product that benefits the recipient phage, cloning of that gene into the would-be recipient phage may augment infection by the recipient phage alone (e.g., Lu & Collins, 2007). There are two caveats to this approach, however. First, cloned genes may be selected against, even if the cloned genes augment infection (Gladstone, Molineux & Bull, 2012); thus the engineered phage may be evolutionarily unstable. Second, processing of the transgene product may depend on its genomic background, such that the gene product does not function properly when cloned by itself (e.g., Firozi et al., 2010).

The perspective here has been one of phage bacterial dynamics—improved bacterial killing by phages. Phage therapy success may depend heavily on phages altering interactions with the immune system, such as by stripping protective surfaces from the bacteria (e.g., Mushtaq et al., 2004). The concept of synergy may be extended to these alternative mechanisms of treatment success, whereby two phages improve treatment outcomes better than either alone. However, identifying this form of synergy *a priori* will no doubt prove challenging, as there is no obvious *in vitro* test for a phage-enhanced immune response.

## Methods

### Media and growth conditions

LB broth was 10 g Bacto tryptone, 10 g NaCl, 5 g Bacto yeast extract per L. T broth was 10 g Bacto tryptone, 5 g NaCl per L. M9 glucose was 47.8 mM Na_2_HPO_4_, 22 mM KH_2_PO_4_, 8.5 mM NaCl, 1.87 mM NH_4_Cl, 1 mM MgS0_4_, 0.1 mM CaCl_2_ with 0.2% glucose per liter (Difco™M9 Minimal Salts, BD). Plates used media with 1.5% Bacto agar. Determinations of phage titers used plates overlaid with soft agar (0.7% Bacto agar) containing a suitable density of hosts. Bacteria were mixed with phage and soft agar, poured on plates, and incubated. J8-65 was titered on either IJ2308 in M9 glucose overnight at 37 °C or on MG1655/T7R in LB overnight at room temperature.

### Strains

Strain information is given in Table 2. J8-65 was obtained from Moscow, ID sewage on M9 glucose plates using the KEIO *lon* bacterium as host (JW0429, KEIO plate 7, H-10). Plaques were large and clear, but it was subsequently realized that they carried two phages, one of which was J8-65; the identity of the second phage was likely T7, but its identity was pursued only superficially once J8-65 was isolated in pure form. The genomic sequence of J8-65 indicates that it is a member of the *ϕ*KMV group of podovirids, with a tailspike 87% identical in protein sequence to NST1 colanidase (Firozi et al., 2010) (GenBank HM214492).

Host IJ2308 was obtained as a visibly mucoid colony growing in a streak of the orignal, two-phage combination plated on a lawn of the KEIO *lon* mutant JW0249 (LB media). Host SG12078 was obtained from S Gottesman, IJ2307 from D Scholl.

**Table 2:** Strain information.

Notation	Genotype/phenotype	Use	Ref
**Bacteria**			
IJ1133	*E. coli* K-12 Δ*lacX74 thi* Δ(*mcrC-mrr*)102::Tn10	Host for plating T7 while avoiding growth of phage J8-65.	^a^
IJ2308	Mucoid mutant of KEIO JW0429 (*lon*)	Host for growth of both T7 and J8-65	^b^
MG1655/T7-R	*E. coli* K-12 MG1655 resistant to T7	Host for plating J8-65 while avoiding growth of T7	^c^
IJ2307	MG6155 T7-R (mucoid)	Mucoid host for testing generality of growth phenotypes of J8-65	^d^
SG12078	C600 (*thr-1 leuB6 tonA21 lacY1 supE44 rfbD1 thi-1* *mcrA e14-*) *rcs137*(constit.) *zei10*::cat	Mucoid host for testing generality of growth phenotypes of J8-65	^e^
**Phage**			
J8-65	*ϕ*KMV-like phage	Colanidase producing phage as donor to T7	^c^
T7^+^	Wild-type(Genbank V01146)		^f^
T7-61	IJ1133-adapted T7	Used as synergy recipient with J8-65	^g^

**Notes.**

a (Garía & Molineux, 1995).

b (Baba et al., 2006).

c This paper.

d (D Scholl, 2013, unpublished data).

e (S Gottesman, 2013, unpublished data).

f (Dunn & Studier, 1983).

g (Heineman & Bull, 2007).

### Plaque phenotypes in different environments

J8-65 was plated on 3 different *E. coli* mucoid K-12 strains (IJ2307, IJ2308, SG12078), at 3 temperatures (37°, 30°, and 24°), each on 3 different media (LB, tryptone broth, M9 glucose supplemented with L-threonine, L-leucine and vitamin B1). Plates were incubated overnight at the respective temperature. Plaque morphology and halo morphology varied greatly with plating condition. On LB or tryptone media in the absence of glucose, J8-65 usually forms plaques with small clear centers (1 mm) but under some conditions they are larger (2–3 mm). Halos typically range from undetectable to 2 mm on tryptone or LB, but were generally more pronounced on media containing glucose. No consistent patterns were evident with respect to temperature or media across all three hosts. The only conditions that consistently gave large, slightly turbid plaques with large halos similar to those in Fig. 1 were with IJ2308 on M9 glucose at 30°and 37°. These conditions (37°) are the ones used here for testing synergy, as they seem maximally suited for IJ2308 to act as a donor phage.

### Cell viability assay in presence of phages

IJ2308 cells, or IJ2308 with approximately 4 × 10^5^ total phage (J8-65 alone, T7-61 alone, or both together), were spread on M9 glucose plates (without soft agar) and grown overnight at 37 °C. The mature growths of phage and bacteria were scraped into 0.85% NaCl, centrifuged for 10 min at 3,500 g, resuspended and centrifuged again and ultimately resuspended in 5 mL 0.85% NaCl. Cells were stained with the LIVE/DEAD BacLight Bacterial Viability Kit (Life Technologies) according to the manufacturer’s instructions.

Stained cells were subjected to flow cytometric analysis using an Accuri C6 Flow Cytometer with the standard laser set and C6 Sampler attachment. Data acquisition was handled by the CFlow Sampler software and subsequent gating was done with a custom GNU R script using the *flowCore* and *flowViz* Bioconductor packages (Fig. S1) (Gentleman et al., 2004). The final data were exported into LibreOffice Calc for further analysis. Thresholds for live and dead cells were established by controls using ethanol-killed cells (dead) and untreated cells (live). As expected, a majority of cells were scored as live in all treatments; the main difference among treatments was in the density of total cells recovered.

### Colanic acid degradation assay

IJ2308 cells, or IJ2308 with approximately 4 × 10^5^ of phage J8-65, were spread on M9 glucose plates (without soft agar) and grown overnight at 37 °C. The mature growths of phage and bacteria were scraped into 1 mL of 0.85% NaCl, vortexed and pelleted. The supernatant containing free colanic acid was transferred to a microfuge tube and acetone precipitated. As previously described (Sutherland, 1971), the fluffy precipitate floating in solution is made of long colanic acid polymer. This suspended precipitate was captured without centrifugation, transfered to an empty tube, air dried and weighed. Five replicates were done for each treatment.

### Sequencing and assembly

Genomic DNA was extracted from J8-65 by phenol extraction followed by isopropanol precipitation. Sequencing was done by Illumina MiSeq by the Genome Sequencing and Analysis Facility at UT Austin. The genome sequence was assembled using SSAKE v3.8 (Warren et al., 2007), and has been deposited in Genbank KM247287. Assembly was done on the Lonestar cluster at the Texas Advanced Computing Center.

### Graphics

Figures were drawn in R (R Development Core Team, 2012).

### Simulations

Differential equations were evaluated numerically in the program Berkeley Madonna (v. 9.0.118 beta) with a step size of 10^−3^ and method Runge–Kutta 4. The numerical output was transferred to R for presentation.

## Supplemental Information

10.7717/peerj.590/supp-1 Figure S1 Representative example of live cell counting by flow cytometry (A–D) Scatter plots of propidium iodide (PI) versus SYTO-9 fluorescent signal. A vertical gate (red line) was used to separate live cells from dead cells. The percentage of live cells in each population is indicated in each panel. (A) IJ2308 cells alone. (B) IJ2308 cells with phage J8-65 only. (C) IJ2308 cells with T7-61 alone. (D) IJ2308 cells with both phages together. Axes are log_10_ of mean fluorescence.

## Additional Information and Declarations

### DNA Deposition

The following information was supplied regarding the deposition of DNA sequences: Genbank KM247287.
