# Supplementary figures and images for: Synergy as a rationale for phage therapy using phage cocktails
Source: PeerJ. 2014 Sep 25;2:e590. doi: 10.7717/peerj.590 (PMC4179555; doi:10.7717/peerj.590)

**A. IJ2308**

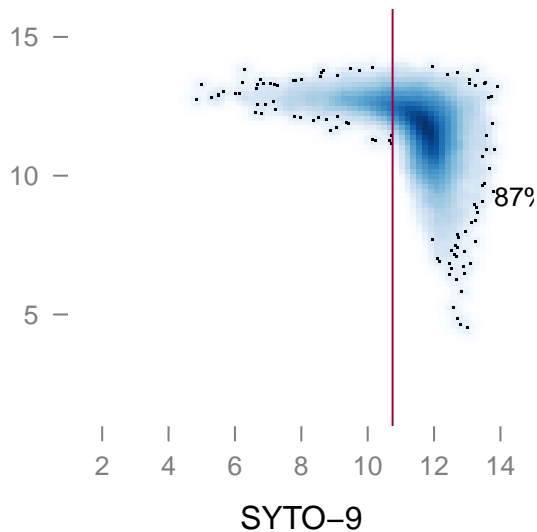

**B. J8-65**

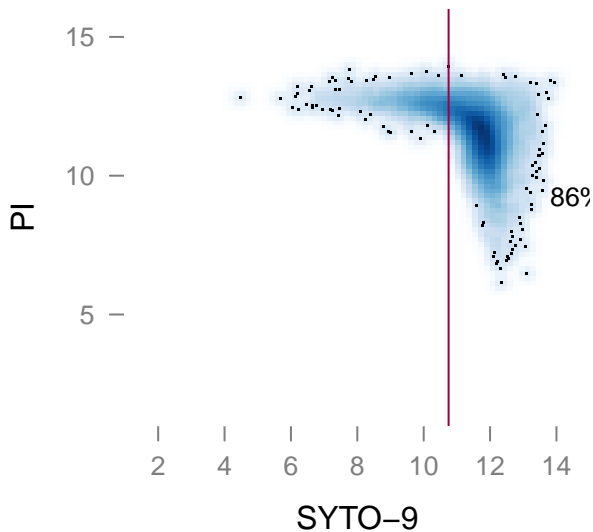

**C. T7-61**

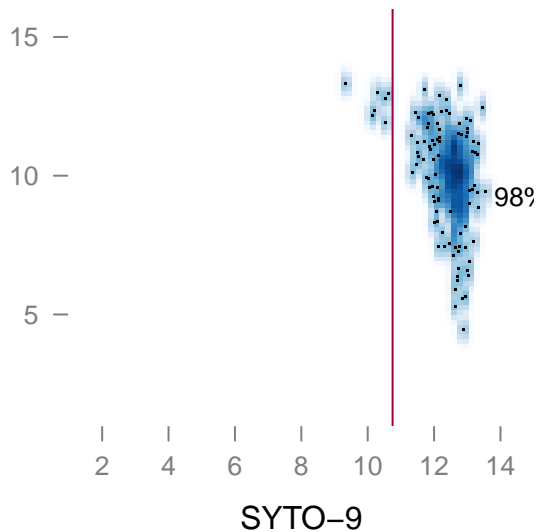

**D. J8-65 + T7-61**

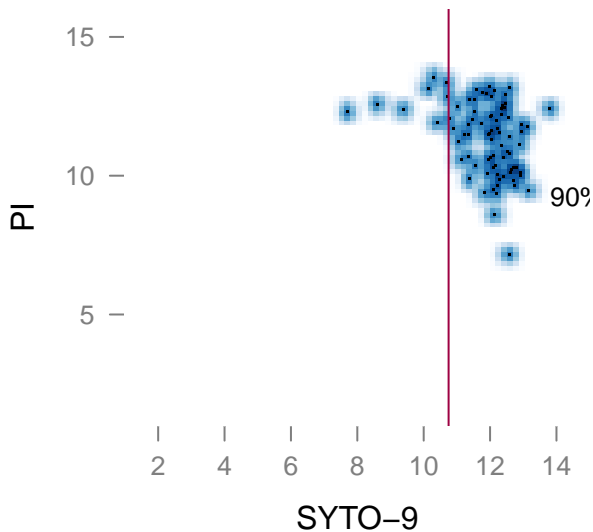

Supplement: Figure S1 — (A–D) Scatter plots of propidium iodide (PI) versus SYTO-9 fluorescent signal. A vertical gate (red line) was used to separate live cells from dead cells. The percentage of live cells in each population is indicated in each panel. (A) IJ2308 cells alone. (B) IJ2308 cells with phage J8-65 only. (C) IJ2308 cells with T7-61 alone. (D) IJ2308 cells with both phages together. Axes are log10 of mean fluorescence. [file peerj-02-590-s001.pdf]
